# New Medical Journals

**Published:** 1866-06

**Authors:** 


### New Medical Journals.

*The Medical and Surgical Monthly*, Memphis, Tenn. Edited by Drs. Ramsey, Saunders, Willett and White.

*The Atlantic Medical and Surgical Journal.* Edited by Dr. J. G. Westmoreland and Dr. W. F. Westmoreland.

Messrs. Ticknor & Fields, of Boston, have placed upon our table their welcome serials for May. The established reputation of the *Atlantic Monthly* leaves no room for additional praise. *Our Young Folks* is the most readable of all the periodicals issued designed for the rising generation; and *Every Saturday* is a weekly which contains, in the form of judicious selections, the cream of current literature.

